# SARS-CoV-2-Host Chimeric RNA-Sequencing Reads Do Not Necessarily Arise From Virus Integration Into the Host DNA

**DOI:** 10.3389/fmicb.2021.676693

**Published:** 2021-06-02

**Authors:** Anastasiya Kazachenka, George Kassiotis

**Affiliations:** ^1^Retroviral Immunology, The Francis Crick Institute, London, United Kingdom; ^2^Department of Infectious Disease, St Mary’s Hospital, Imperial College London, London, United Kingdom

**Keywords:** SARS-CoV-2, integration, reverse transcription, retroelements, RNA-sequencing

## Abstract

The human genome bears evidence of extensive invasion by retroviruses and other retroelements, as well as by diverse RNA and DNA viruses. High frequency of somatic integration of the RNA virus severe acute respiratory syndrome coronavirus 2 (SARS-CoV-2) into the DNA of infected cells was recently suggested, based on a number of observations. One key observation was the presence of chimeric RNA-sequencing (RNA-seq) reads between SARS-CoV-2 RNA and RNA transcribed from human host DNA. Here, we examined the possible origin specifically of human-SARS-CoV-2 chimeric reads in RNA-seq libraries and provide alternative explanations for their origin. Chimeric reads were frequently detected also between SARS-CoV-2 RNA and RNA transcribed from mitochondrial DNA or episomal adenoviral DNA present in transfected cell lines, which was unlikely the result of SARS-CoV-2 integration. Furthermore, chimeric reads between SARS-CoV-2 RNA and RNA transcribed from nuclear DNA were highly enriched for host exonic, rather than intronic or intergenic sequences and often involved the same, highly expressed host genes. Although these findings do not rule out SARS-CoV-2 somatic integration, they nevertheless suggest that human-SARS-CoV-2 chimeric reads found in RNA-seq data may arise during library preparation and do not necessarily signify SARS-CoV-2 reverse transcription, integration in to host DNA and further transcription.

## Introduction

Viruses hijack the host cell to replicate their RNA or DNA genomes and create progeny virions. An extreme form of viral parasitism is the integration of a viral genome DNA copy into the host cell DNA (Burns and Boeke, 2012; Feschotte and Gilbert, 2012). Although diverse classes of RNA viruses create a complementary DNA (cDNA) copy through reverse-transcription of their genomes during their life cycle, integration into the host DNA is a characteristic obligatory step for retroviruses, as well as for endogenous retroelements (Coffin et al., 1997; Burns and Boeke, 2012; Feschotte and Gilbert, 2012).

The machinery that mediates reverse transcription and integration of the retroviral and endogenous retroelement genomes can also use alternative RNA templates, creating genomic cDNA copies of the latter. For example, mammalian apparent long terminal repeat (LTR)-retrotransposons (MaLRs) rely on endogenous retroviruses (ERVs) for their reverse-transcription and integration. Similarly, short interspersed nuclear elements (SINEs), including *Alu* elements, rely on long interspersed nuclear elements (LINEs) for their reverse transcription and integration (Coffin et al., 1997; Burns and Boeke, 2012; Feschotte and Gilbert, 2012).

The reverse transcriptase and endonuclease activity of LINEs, carried out by the ORF2p protein, can also mediate reverse transcription and integration of unrelated viral and non-viral RNAs (Klenerman et al., 1997; Esnault et al., 2000; Buzdin, 2004). Indeed, the human genome contains DNA copies of distinct RNA and DNA viruses (Blinov et al., 2017), as well as numerous retrogenes and pseudogenes (Baertsch et al., 2008; Richardson et al., 2014; Staszak and Makałowska, 2021), highlighting the possible, albeit infrequent, reverse transcription and integration of non-retroviral RNA into the host genome.

Recent studies reported a high frequency of reverse transcription and integration of severe acute respiratory syndrome coronavirus 2 (SARS-CoV-2) RNA in infected cells (Zhang et al., 2020; Ying et al., 2021), with implications for diagnostic detection of SARS-CoV-2 nucleic acids by RT-qPCR and for viral antigen persistence. These findings were partly based on the identification of chimeric reads between viral and human RNA in next-generation RNA-sequencing (RNA-seq) data (Zhang et al., 2020; Ying et al., 2021). Here, we examined the potential source of such chimeric reads and found that they are more likely to be a methodological product, than the result of genuine reverse transcription, integration, and expression.

## Materials and Methods

### RNA-seq Analysis

Public RNA-seq datasets under the accession numbers GSE147507 (Blanco-Melo et al., 2020), GSE150316 (Desai et al., 2020), and GSE151803 (Han et al., 2021) were downloaded from NCBI Gene Expression Omnibus (GEO) server. Adapter and quality trimming were conducted using Trimmomatic v0.36 (Bolger et al., 2014). Quality of sequencing reads was assessed by FastQC v0.11.5. The resulted reads were aligned to the merged GRCh38/hg38 genome (including alternative and random chromosome sequences) and SARS-CoV-2 NC_045512v2 genome using STAR v2.7.1 aligner (Dobin et al., 2013). GENCODE v29 basic version and wihCor1 NCBI genes were used for human and SARS-CoV-2 gene annotations, respectively.^1^ Chimeric reads were called using STAR parameters as used in prior reports (Zhang et al., 2020). Minimal overhang for a chimeric junction (--chimJunctionOverhangMin) and minimal length of chimeric segment length (--chimSegmentMin) parameters were set as 50 for analysis of singled-end RNA-seq datasets (GSE147507; GSE151803) and as 25 for analysis of paired-end RNA-seq dataset (GSE150316). Gene expression was calculated by FeatureCounts (part of the Subread package v1.5.0; Liao et al., 2014) and normalized with DESeq2 v1.22.1 within R v3.5.1 (Love et al., 2014). The Integrative Genomics Viewer (IGV) v2.5.3 was used to visualize aligned non-chimeric and chimeric reads (Robinson et al., 2011). BLASTN+ v2.3.0 was used to align mitochondrial RNA-nuclear RNA (mtRNA-nRNA) chimeric reads to identify mitochondrial and nuclear aligning sequences within the reads (Camacho et al., 2009). Reads containing highly homologous sequences to mitochondrial and nuclear genomes simultaneously were removed from analysis. Viral-host chimeric reads were aligned to SARS-CoV-2 and human reference genomes using the same method to quantify overlapping regions between viral and human genome aligning parts of the reads.

## Results

### Human-SARS-CoV-2 Chimeric Reads in RNA-seq Libraries of SARS-CoV-2 Infected Cell Lines

Chimeric reads between human and SARS-CoV-2 RNA have been identified in RNA-seq data from infected cells in two recent studies (Zhang et al., 2020; Ying et al., 2021), presumed to be transcribed from reversed transcribed SARS-CoV-2 RNA integrated into the host DNA. To confirm these findings and exclude alternative origins of virus-host chimeric reads, we analyzed public RNA-seq datasets of cells infected with unrelated RNA viruses or SARS-CoV-2, and lung samples from a coronavirus disease 2019 (COVID-19) patient and a healthy control, using a standard pipeline, also used in the previous studies (Zhang et al., 2020; Ying et al., 2021).

To this end, we used RNA-seq from normal human bronchial epithelial (NHBE) cells, A549 cells that do not normally express *ACE2*, encoding the cellular receptor for SARS-CoV-2, and A549 cells overexpressing *ACE2* from an adenoviral vector, and Calu3 cells that naturally express *ACE2* (GSE147507). These cells were infected with SARS-CoV-2 or with other respiratory viruses, including human parainfluenza virus type 3 (HPIV3), influenza A virus (IAV), IAV lacking the antiviral NS1 gene (IAVdNS1), and respiratory syncytial virus (RSV), or were left uninfected, and were subsequently subjected to RNA-seq (Blanco-Melo et al., 2020). In addition, we used RNA-seq data (GSE147507) from a lung sample from a COVID-19 patient and a lung sample from a healthy uninfected individual (Blanco-Melo et al., 2020). The latter were supplemented with RNA-seq data (GSE150316) from lung samples from a further five COVID-19 patients and one healthy uninfected individual, each providing multiple samples (Desai et al., 2020). Lastly, we used RNA-seq from lung samples from another three COVID-19 patients (GSE151803), produced similarly to those described in the contributors’ report (Han et al., 2021).

**Figure 1:**
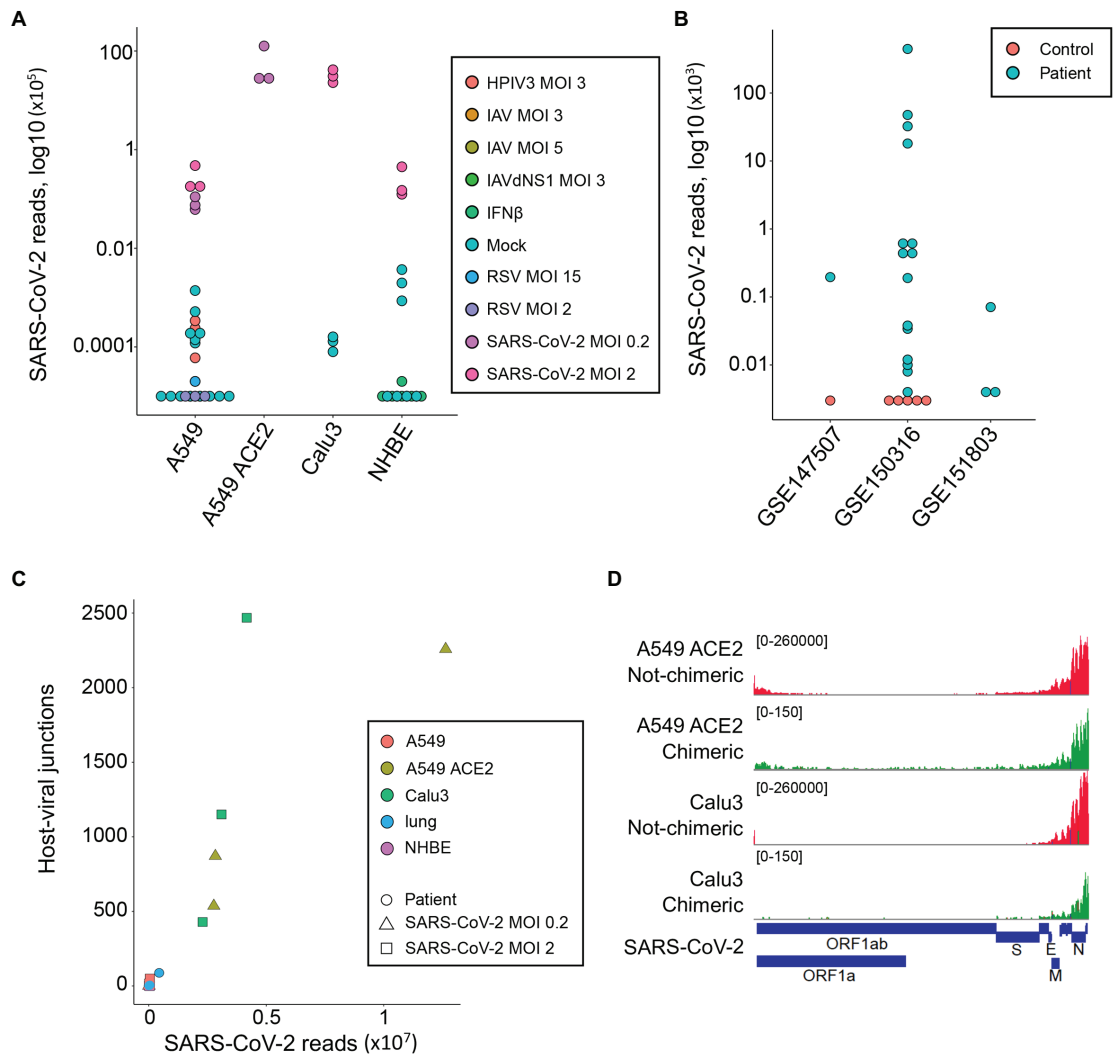
Detection of human-severe acute respiratory syndrome coronavirus 2 (SARS-CoV-2) chimeric reads in RNA-sequencing (RNA-seq) data. **(A)** Number of non-chimeric reads uniquely aligning to SARS-CoV-2 genome in RNA-seq data (GSE147507) from parental A549 cells, A549 cells overexpressing ACE2 (A549 ACE2), Calu3 cells, and normal human bronchial epithelial (NHBE) cells. The cells were infected or not (Mock) with human parainfluenza virus type 3 (HPIV3), influenza A virus (IAV), IAV lacking the antiviral NS1 gene (IAVdNS1), respiratory syncytial virus (RSV), or SARS-CoV-2, at different multiplicities of infection (MOIs), or treated with recombinant IFNβ (IFNβ). **(B)** Number of non-chimeric reads uniquely aligning to SARS-CoV-2 genome in RNA-seq data from coronavirus disease 2019 (COVID-19) patient lung samples and healthy uninfected control lung samples, from the indicated studies. **(C)** Number of human-SARS-CoV-2 junctions plotted against non-chimeric SARS-CoV-2-mapping reads in the same samples. **(D)** Alignment of human-SARS-CoV-2 chimeric and non-chimeric RNA-seq reads from SARS-CoV-2 infected A549 ACE2 and Calu3 cells across the SARS-CoV-2 genome, visualized on integrative genomics viewer (IGV).

As expected, reads mapping to the SARS-CoV-2 genome were readily found in samples infected with this virus (median = 44,922, range 6,130-12,636,376; Figure 1A). A549 cells overexpressing *ACE2* (A549 ACE2 cells) and Calu3 cells showed the highest number of viral reads, with parental A549 cells and NHBE cells showing lower read numbers (Figure 1A). Minimal numbers of SARS-CoV-2-mapping reads (median = 0, range 0–370) were identified in uninfected cell lines or those infected with unrelated viruses (Figure 1A). Similarly, no SARS-CoV-2-mapping reads were identified in lung samples from uninfected individuals, except one where three reads were identified, whereas the number of SARS-CoV-2-mapping reads varied considerably between lung samples taken from COVID-19 patients (Figure 1B), consistent with previously observed heterogeneity (Desai et al., 2020).

In agreement with earlier reports (Zhang et al., 2020; Ying et al., 2021), we identified host-viral junctions in SARS-CoV-2 infected cell lines, in direct proportion with the number of SARS-CoV-2 non-chimeric reads (Figure 1C). Supported human-SARS-CoV-2 chimeric reads constituted between 0.002 and 0.14% of all SARS-CoV-2-mapping reads found in infected cell lines, in line with the proportion of chimeric reads reported in earlier studies (Zhang et al., 2020; Ying et al., 2021). Reads with chimeric junctions were far rarer in lung samples, in proportion with SARS-CoV-2 non-chimeric reads, with between 2 and 93 chimeric reads in five lung samples from two COVID-19 patients. Also in agreement with earlier studies, the viral parts of human-SARS-CoV-2 chimeric reads preferentially aligned to the 3' end of the viral genome, mirroring general transcriptional activity of the viral genome (Figure 1D). Thus, human-SARS-CoV-2 chimeric reads are detectable in RNA-sed data, with the viral part donated more frequently from the highest expressed 3' end of the viral genome.

### Non-canonical Origin of the Human Part in Human-SARS-CoV-2 Chimeric Reads

**Figure 2:**
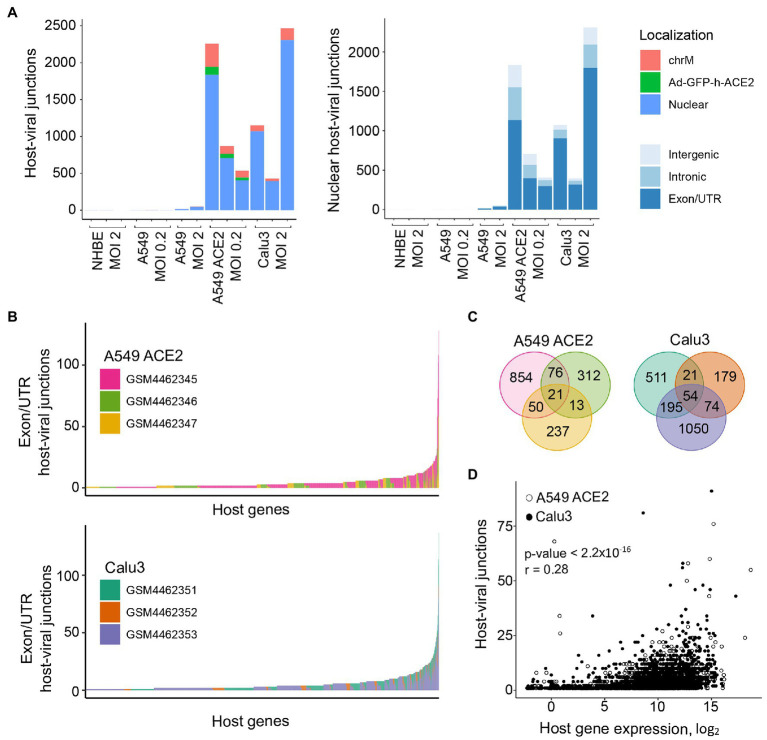
Characteristics of human sequence part of human-SARS-CoV-2 chimeric reads. **(A)** *Left*, number of chimeric junctions identified in the indicated samples, according to the origin of the human sequence part (chrM, mitochondrial DNA; Ad-GFP-h-ACE2, ACE2-encoding adenoviral vector episomal DNA, Nuclear, nuclear DNA). *Right*, number of chimeric junctions where the human sequence part aligns to nuclear human DNA, according to position relative to annotated genes and exons. Each bar presents each of the triplicate samples. **(B)** Number of chimeric junctions located within coding regions of individual human nuclear genes in A549 ACE2 and Calu3 RNA-seq datasets. Nuclear genes donating the human sequence part are plotted on *x*-axis and each bar represents an individual gene, color-coded according to the triplicate sample in which it was found. **(C)** Overlap of host nuclear genes found in chimeric reads between the triplicate A549 ACE2 and Calu3 samples. **(D)** Correlation between the number of human-SARS-CoV-2 chimeric reads and the level of human donor gene expression.

We next examined the possible location of the human sequence part found in human-SARS-CoV-2 chimeric reads along the human genome. Of all chimeric reads identified in SARS-CoV-2 infected A549 ACE2 cells, between 12.2 and 17.7% were formed between human mitochondrial and viral RNA (Figure 2A). In SARS-CoV-2 infected Calu3 cells, mitochondrial RNA-SARS-CoV-2 chimeric reads comprised between 6.5 and 7.2% of total chimeric reads. Between 4.8 and 6.7% of chimeric reads in A549 ACE2 cells aligned to the ACE2 gene (Figure 2A). However, no ACE2-SARS-CoV-2 chimeric reads were found in other cell lines, including A549 cells. ACE2 overexpression in A549 ACE2 cells was achieved *via* transfection with an ACE2-expressing adenoviral vector (Blanco-Melo et al., 2020). As ACE2 in A549 ACE2 cells is transcribed from the adenoviral vector, chimeric ACE2-SARS-CoV-2 RNA-seq reads found in these cells would have required integration into the episomal adenoviral vector. Together, human-SARS-CoV-2 chimeric reads where the human part was donated by mitochondrial RNA or ACE2 RNA transcribed from the episomal adenoviral vector accounted for approximately a quarter of all chimeric reads (Figure 2A).

The remaining human-SARS-CoV-2 chimeric reads aligned to nuclear genome. Of these, between 56.6 and 84.3% were located within annotated coding exons or untranslated regions (UTRs), whereas chimeric reads aligning to introns or intergenic regions were far fewer (Figure 2A). Notably, certain host genes contributed disproportionally to chimeric reads (Figure 2B). A549 ACE2 and Calu3 cells, 21 and 54 genes, respectively donated the human part of chimeric reads found in all three replicates of each cell line, and 139 and 290 genes, respectively contributed to chimeric reads in two of the replicates (Figure 2C). Host genes with higher contribution to chimeric reads also tended to be expressed at higher levels (Figure 2D). The recurrent contribution (between 14 and 45%) of the same highly expressed genes to chimeric reads in independent replicates of A549 ACE2 and Calu3 cell infection with SARS-CoV-2 indicates that the process that creates these chimeric reads was efficiently repeated in each replicate.

### Alternative Mechanisms Creating Chimeric Reads in RNA-seq Libraries

In addition to reverse transcription and integration of viral RNA, followed transcription of the integrated copy, several alternative mechanisms might explain formation of chimeric RNA, such as genomic rearrangements, trans-splicing, or transcriptional slippage (Yang et al., 2013). However, joining of transcripts from separate chromosomes or between host and viral RNA remains theoretical. An alternative mechanism for formation of inter-chromosomal chimeric reads in RNA-seq libraries has also been proposed (Li et al., 2009; Peng et al., 2015; Xie et al., 2016). This involves consecutive reverse transcription reactions, were cDNA sequences created during one reverse transcription reaction may prime reverse transcription of an unrelated RNA sequence through complementarity provided by small homologous sequences (SHS; Li et al., 2009; Peng et al., 2015; Xie et al., 2016). The generation of artificial chimeric sequences *via* consecutive reverse transcription reactions is indirectly supported by the presence of mtRNA-nRNA fusions in public expression sequence tags (ESTs) databases. The spatial separation of mitochondrial and nuclear DNAs negates transcriptional slippage or trans-splicing, leaving consecutive reverse transcription reactions through SHS-mediated priming as a possible cause.

**Figure 3:**
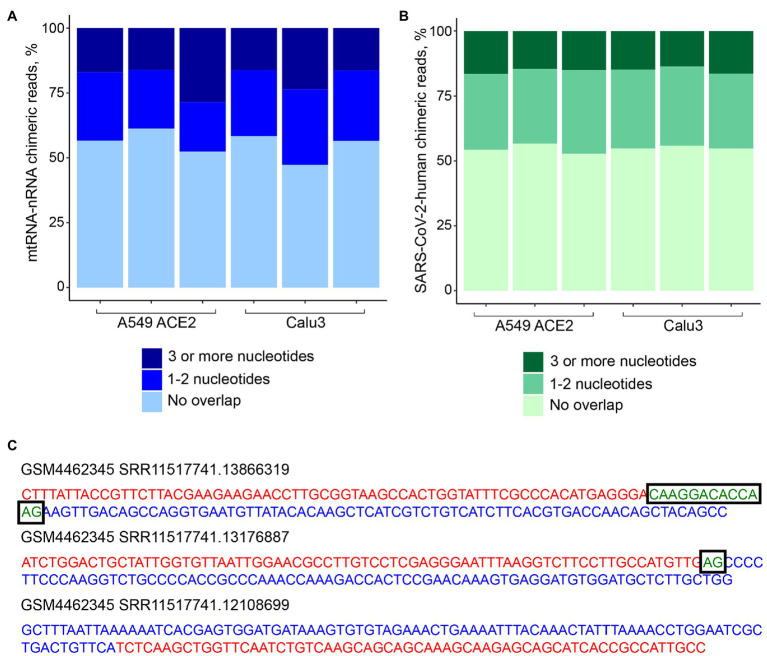
Sequence homology at the junctions of human-SARS-CoV-2 chimeric reads. **(A)** Sequence overlap between mitochondrial and nuclear DNA in mitochondrial RNA-nuclear RNA (mtRNA-nRNA) chimeric reads. Each bar presents each of the triplicates of SARS-CoV-2 infected A549 ACE2 and Calu3 cells. **(B)** Sequence overlap between SARS-CoV-2 genomic RNA and the human genome in human-SARS-CoV-2 chimeric reads in the same samples. **(C)** Representative examples of human-SARS-CoV-2 chimeric reads with 13, 2, and 0 nucleotide overlap. SARS-CoV-2 and human genomic sequences are shown in red and blue letters, respectively. Overlapping sequences are shown in boxed green letters.

To address the possibility that human-SARS-CoV-2 chimeric reads were formed *via* SHS-mediated priming during RNA-seq library construction, we first searched for mtRNA-nRNA chimeric reads, in order to assess the frequency of SHS at the junction of artifactual chimeric reads. Between 16 and 28% of analyzed mtRNA-nRNA junctions exhibited an overlap of three or more nucleotides between mitochondrial and nuclear sequences (Figure 3A). We next looked for similar SHS across junctions of human-SARS-CoV-2 chimeric reads (Figures 3B,C). Between 14 and 16% of human-SARS-CoV-2 junctions had three or more overlapping nucleotides, which was comparable with their proportion in mtRNA-nRNA junctions (Figures 3A,B). Thus, SHS-mediated priming may be responsible for at least a fraction of human-SARS-CoV-2 chimeric reads detected in RNA-seq libraries.

## Discussion

The pandemic caused by SARS-CoV-2 that currently continues to spread globally (Hu et al., 2020), highlighted the need for deeper understanding of its interaction with the human host. The possible genomic integration of SARS-CoV-2 nucleic acids (Zhang et al., 2020; Ying et al., 2021) would have significant implications for host-viral interaction.

The somatic integration of a DNA copy of the RNA virus lymphocytic choriomeningitis virus (LCMV) in the murine host can provide a source of persistent antigen for the immune system (Klenerman et al., 1997). Similarly, persistence of somatically integrated SARS-CoV-2 DNA copies with coding potential could prolong presentation of viral antigens. However, analyses of intestinal biopsies several months after recovery from COVID-19, indicated the presence of SARS-CoV-2 RNA, as well as presumptive SARS-CoV-2 virions, consistent with on-going replication (Gaebler et al., 2021). Therefore, detection of persistent viral antigen may not necessarily indicate somatic SARS-CoV-2 integration.

Detection of chimeric reads between SARS-CoV-2 RNA and human RNA could also be indicative of somatic SARS-CoV-2 integration. Since detection of such chimeric reads in RNA-seq data would require transcription of the somatic integration, it would likely underestimate the total number of integrations. The high frequency of expressed somatic SARS-CoV-2 integrations reported (Zhang et al., 2020; Ying et al., 2021) was, therefore, unexpected. However, the majority of chimeric human-SARS-CoV-2 RNA reads may have a different origin. We identified chimeric reads between SARS-CoV-2 RNA and mitochondrial RNA, which were unlikely to have resulted from transcription of SARS-CoV-2 DNA copies integrated into mitochondrial DNA. If these reads were the result of SARS-CoV-2 integration into mitochondrial DNA, this would require mitochondrial import of viral cDNA and of components of canonical non-homologous end joining (NHEJ) process. While low levels of NHEJ had been reported in mitochondria, no evidence of viral DNA retrotransposition into the mitochondrial genome has yet been reported. Similarly, we identified chimeric reads between SARS-CoV-2 and RNA transcribed from the adenoviral vector used to overexpress *ACE2*, in target cells (Blanco-Melo et al., 2020), which would have necessitated integration of SARS-CoV-2 DNA copies in episomal adenoviral DNA. The finding that up to 24% of chimeric reads were formed between SARS-CoV-2 RNA and RNA transcribed from mitochondrial DNA or episomal adenoviral DNA suggested similarly artifactual generation of the remaining reads.

Chimeric reads between nuclear DNA-transcribed RNA and SARS-CoV-2 RNA involved host genes expressed at higher than average level. This correlation may have resulted from more probable detection of the higher expressed, than lower expressed genuine chimeric fragments. Alternatively, it could result from more frequent fortuitous joining, such as during RNA-seq library preparation for example, of SARS-CoV-2 RNA reads with the most abundant host gene RNA reads in the library. In support of the latter possibility, a substantial proportion of chimeric reads displayed complementarity, often over 10 nucleotides, in the joining region. Moreover, the substantially higher contribution of exonic than intronic or intergenic host sequences to human-SARS-CoV-2 chimeric reads is consistent with formation during RNA-seq library preparation, where exonic sequences are overrepresented relative to intronic or intergenic sequences.

Detection of chimeric reads between SARS-CoV-2 RNA and human RNA is one of several distinct methods previously employed to estimate somatic SARS-CoV-2 integration (Zhang et al., 2020; Ying et al., 2021). Given its dependency on transcription of integrated SARS-CoV-2 cDNA, in addition to the integration step itself, it is likely to be the least sensitive. Direct detection of integrated SARS-CoV-2 cDNA in host genomic DNA, regardless of its expression, was not possible for the datasets used in this study, as whole-genome sequencing data were not available. Accordingly, the data presented here do not rule out the possibility that SARS-CoV-2 RNA can be reverse-transcribed and integrated in the host DNA. Instead, our study examined specifically the extent to which such integration events can be supported by the detection of chimeric reads between SARS-CoV-2 RNA and human RNA. At least at the level that can be determined by RNA-seq data analysis, our findings do not indicate frequent genomic integration and subsequent expression of SARS-CoV-2 RNA, and similar conclusions were reached by independent analysis (Yan et al., 2021).

## Data Availability Statement

Publicly available datasets were analyzed in this study. This data can be found at: https://www.ncbi.nlm.nih.gov/geo/ under the series GSE147507, GSE150316, and GSE151803.

## Author Contributions

AK analyzed the data. AK and GK wrote the manuscript. Both the authors contributed to the article and approved the submitted version.

### Conflict of Interest

The authors declare that the research was conducted in the absence of any commercial or financial relationships that could be construed as a potential conflict of interest.

## Footnotes

^1^http://hgdownload.soe.ucsc.edu/goldenPath/wuhCor1/bigZips/genes/
